# Salicylic acid moonlights beyond defense to guard rice seed longevity

**DOI:** 10.1093/plphys/kiag267

**Published:** 2026-05-06

**Authors:** Neeta Lohani

**Affiliations:** Assistant Features Editor, Plant Physiology, American Society of Plant Biologists; Department of Biotechnology, Thapar Institute for Engineering and Technology, Patiala, Punjab 147004, India

A seed is a biological time capsule, packed with everything a new plant needs to germinate and establish itself. But the molecular cargo inside does not remain intact indefinitely. Seed longevity, the ability to maintain viability during storage, is a critical agronomic trait that directly impacts crop productivity and food security. Over time, reactive oxygen species (ROS) accumulate, membranes lose integrity, and energy metabolism declines, progressively eroding seed viability. Given that rice feeds over one-half of the world's population, understanding the molecular mechanisms that protect seeds against this deterioration is essential for germplasm conservation and reliable crop establishment. While the hormonal and signaling networks governing seed aging have been partially characterized, much of this regulatory landscape remains unexplored.

When it comes to hormonal control of seed longevity, abscisic acid (ABA) has long held center stage. Its signaling components, including ABI3, ABI5, and the PYR/PYL/RCAR–PP2C–SnRK2 cascade, are well-established regulators of seed maturation, desiccation tolerance, and storage stability (Clerkx et al. 2004; Zinsmeister et al. 2016). Salicylic acid (SA), by contrast, is best known as the architect of systemic acquired resistance against pathogens, and its appearances in seed biology have been limited to scattered observations, from rejuvenating aged soybean seeds to boosting vigor under salt stress (Nazari et al. 2020). But with the complete PAL-dependent SA biosynthesis pathway in rice now decoded (Wang et al. 2025), the machinery is in place to ask a more ambitious question. Could this defense hormone be moonlighting as a longevity regulator, quietly coordinating the very pathways that keep seeds viable in storage?

Recently in *Plant Physiology*, Wang et al. (2026) provide compelling evidence that SA is not merely a bystander but a central hormonal hub that integrates multiple signaling pathways to regulate seed longevity in rice. The authors combine multi-omics profiling, CRISPR-Cas9 gene editing, and protein–protein interaction analyses to dissect a regulatory network centered on SA. Their work reveals 9 genes spanning 6 signaling pathways whose coordinated action determines how long a rice seed remains viable.

Wang et al. subjected 3 japonica rice cultivars, ZH11, NJ505, and XF9, to accelerated aging (AA) at 42 °C and 70% relative humidity for 12 d (Fig. 1a). ZH11 seeds maintained significantly higher germination rates and seedling vigor than NJ505 and XF9, establishing it as the AA-tolerant cultivar. ZH11 seeds also accumulated less ROS and contained higher endogenous SA, while ABA levels dropped after AA, a hormonal profile consistent with better stress resilience. To uncover the molecular basis of this tolerance, the authors profiled the transcriptome and widely targeted metabolome of AA and non-AA ZH11 embryos, identifying 8,841 differentially expressed genes and 249 differentially accumulated metabolites. Integrative analysis of these datasets converged on 6 significantly enriched signaling pathways that govern seed longevity, namely oxidative phosphorylation, plant–pathogen interactions, photosynthesis, carbon fixation, ABC transport, and hormone signaling.

**Figure 1:**
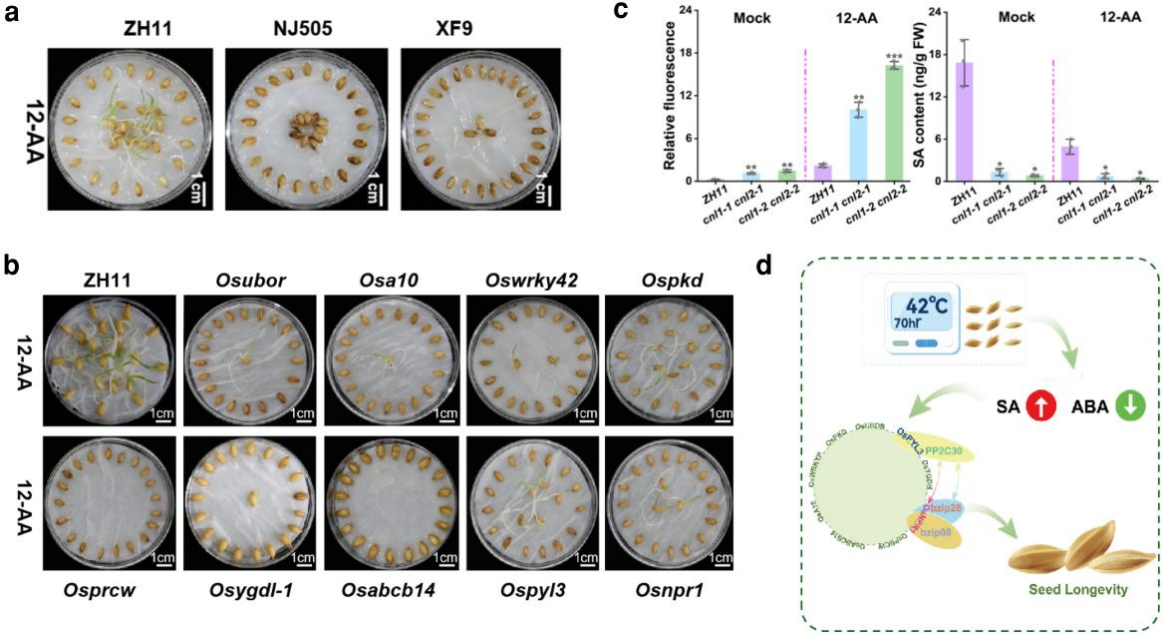
Salicylic acid orchestrates a multi-pathway network for rice seed longevity. a) Seeds of 3 *japonica* rice cultivars (ZH11, NJ505, and XF9) after 12 d of accelerated aging (12-AA), illustrating the superior AA tolerance of ZH11 relative to the aging-sensitive NJ505 and XF9. b) Seed longevity phenotypes of CRISPR-Cas9 knockout mutants for 9 key genes (*Osubor*, *Osa10*, *Oswrky42*, *Ospkd*, *Osprcw*, *Osygdl-1*, *Osabcb14*, *Ospyl3*, and *Osnpr1*) compared with wild-type ZH11 after 12-AA, demonstrating that disruption of any single gene markedly reduces seed viability. c) Total ROS levels (left) and endogenous SA content (right) in wild-type ZH11 and the SA biosynthesis double mutants *cnl1-1cnl2-1* and *cnl1-2cnl2-2* under non-AA (Mock) and 12-AA conditions. Reduced endogenous SA in the *cnl* mutants coincides with elevated ROS, and the differences are amplified by AA, providing direct physiological evidence that the PAL-dependent SA biosynthesis pathway maintains seed longevity. d) Schematic model summarizing how AA triggers a coordinated increase in SA and decrease in ABA, with nine core genes across 6 signaling pathways forming a regulatory network linked through the SA receptor NPR1 and newly identified interacting components (bZIP28, bZIP03, PP2C30) to maintain seed longevity. Figure adapted from Wang et al. (2026).

What makes this study particularly robust is the systematic genetic validation that followed the omics discovery. Rather than stopping at correlative associations, Wang et al. generated CRISPR-Cas9 knockout mutants for 9 key node genes (*OsUbOR*, *OsA10*, *WRKY42*, *OsPKD*, *OsPRCW*, *OsYGD1-1*, *OsABCB14*, *OsPYL3*, and *OsNPR1*), each representing a different signaling pathway. Strikingly, disruption of any single one of these 9 genes led to markedly reduced seed viability after AA (Fig. 1b), demonstrating that multiple pathways must work in concert to maintain seed longevity. Even genes with low basal expression in seeds, such as the PSII component *OsPRCW*, proved critical, as its mutant exhibited the most dramatically shortened seed lifespan. This observation challenges the assumption that gene expression levels alone predict functional importance.

The central insight of this work is the positioning of SA as the molecular bridge connecting these diverse pathways. The authors demonstrated that all 9 core genes showed differential expression across the 3 cultivars in a pattern consistent with their endogenous SA levels. Notably, SA content in ZH11 seeds was significantly higher than in NJ505 and XF9 under both non-AA and AA conditions, and the expression of SA biosynthesis genes in the PAL pathway (*CNL-1*, *CNL-2*, *OsD2*, *OsD3*) was significantly lower in the AA-sensitive cultivars. Moreover, exogenous SA application revealed a dose-dependent effect: moderate concentrations (5 to 10 µM) rescued seed germination in AA seeds across all 3 varieties, while higher concentrations (20 to 100 µM) progressively inhibited germination despite continued ROS scavenging. This biphasic response is consistent with SA promoting longevity not by maximally suppressing ROS but by maintaining an optimal SA–ROS balance. More compellingly, functional validation in the endogenous system using SA biosynthesis double mutants (*cnl1-1cnl2-1* and *cnl1-2cnl2-2*) further solidified this conclusion, as these mutants showed reduced endogenous SA, elevated ROS, and shortened seed longevity (Fig. 1c).

Beyond establishing SA as a longevity regulator, Wang et al. also identified new molecular players connecting SA and ABA signaling during seed aging (Fig. 1d). On the SA side, the receptor NPR1 interacts with 2 bZIP transcription factors, bZIP03 and bZIP28. On the ABA side, the receptor PYL3 pairs with the phosphatase PP2C30, an interaction not previously reported. Critically, NPR1 also interacts with PP2C30, placing this phosphatase at a convergence point between the 2 pathways. Although these components still await functional validation through mutant analysis, they represent promising candidates for dissecting how SA and ABA coordinate seed longevity.

This study makes several important contributions to our understanding of seed biology. First, it elevates SA from its canonical role in defense to a central position in seed longevity regulation, a paradigm shift supported by multiple independent lines of evidence spanning cultivar comparisons, multi-omics profiling, gene knockouts, SA biosynthesis mutants, and exogenous hormone rescue experiments. Second, the dose-dependent nature of SA's effects provides a nuanced framework for understanding hormonal regulation, moving beyond simple positive-or-negative classifications. Third, the identification of nine validated genetic targets across 6 pathways offers a rich toolkit for breeding programs aimed at improving seed storage stability, a trait of enormous practical importance for rice production, seed banking, and germplasm conservation worldwide.

Several exciting questions emerge from this work. How do the nine core genes interact at the protein level to form an integrated regulatory network? What upstream signals trigger the SA–ROS dosage mechanism during seed storage? Do the newly identified bZIP03/bZIP28 and PP2C30 components show shortened seed longevity when mutated, and how do they modulate SA–ABA crosstalk at the molecular level? Can the SA-responsive genetic architecture identified in ZH11 be leveraged through marker-assisted selection or genome editing to improve seed longevity in elite but AA-sensitive cultivars? As climate change increasingly threatens seed viability during storage in tropical and subtropical regions, the molecular framework established by Wang et al. offers a timely roadmap for breeding crops with enhanced seed storage potential. The defense hormone, it seems, has been quietly moonlighting as a guardian of seed survival all along.

## Related articles in *Plant Physiology*

- Hazra et al. (2025): Apocarotenoids derived from β-carotene cleavage by phytoene synthase protect seed longevity by reducing ROS accumulation and activating an aquaporin signaling component in *Arabidopsis*.
- Huang et al. (2025): A transcriptional cascade of OsDREB1C–OsNAC3–OsGH3.2 modulates rice seed germination through coordinated ABA-dependent auxin signaling, offering molecular breeding targets for direct-seeding cultivation.

## Data Availability

No new data was generated or analyzed in support of this article.
